# The effect of physical activity intervention and nutritional habits on anthropometric measures in elementary school children: the health oriented pedagogical project (HOPP)

**DOI:** 10.1038/s41366-021-00830-5

**Published:** 2021-05-10

**Authors:** Nandu Goswami, Irhad Trozic, Maren Valand Fredriksen, Per Morten Fredriksen

**Affiliations:** 1 Physiology Division, Otto Loewi Centre of Research in Vascular Biology, Immunity and Inflammation, Medical University of Graz, Graz, Austria; 2 Alma Mater Europeaea ECM Maribor, Slovenia, Norway; 3 School of Health Sciences, Kristiania University College, Oslo, Norway

**Keywords:** Paediatrics, Risk factors

## Abstract

**Background:**

Overweight and obesity are a burden of the modern world that requires urgent action. Prevention has proven to be efficient in the fight against overweight and obesity. As many children gain excessive weight at an early age, intervention during school years are important. While daily physical activity (PA) is known to have an influence on overweight and obesity prevention, the importance of a healthy lifestyle, including dietary habits, should not be underestimated. The aim of this study was to assess how a combination of daily PA and healthy/unhealthy diet affect the anthropometric measures of 4th graders in Norway.

**Methods:**

The Health Oriented Pedagogical Project (HOPP) is a longitudinal intervention in primary school children, which includes increased amount of daily physical activity during teaching– active learning. Assessed were weight, muscle and bone mass, as well as fat mass, using a bio-impedance Tanita scale. A dietary survey, Ungkost 2000, with 18 multiple-choice questions was used to evaluate the overall nutrition characteristics of the children.

**Results:**

Between 2015 and 2018, a total of 917 (intervention group *n* = 614, control group *n* = 303) 4th graders from nine different schools from the south–east part of Norway participated. We observed that daily PA and a regular healthy diet increases—while an unhealthy diet decreases—muscle and bone mass despite daily PA. Daily PA appears to counteract some of the effects of an unhealthy diet on weight and fat mass. In addition, daily PA and a regular intake of fruits and berries lowers weight and fat mass in children with overweight.

**Conclusion:**

Combination of daily 45-minute of PA and a healthy diet leads to reductions in body weight and incease in muscle and bone mass in elementary school children.

## Introduction

In recent decades, increased incidence in lifestyle related morbidities such as diabetes and cardiovascular diseases has led to reductions in the quality of life in individuals and increased healthcare-related costs [1]. It is important to address the roots of these morbidities, as unhealthy lifestyle, physical inactivity and an unhealthy diet continue to increase globally [2]. Lack of physical activity (PA) combined with increased intake of an unhealthy diet with a high caloric value negatively affects body composition and health status [3]. A consequence of an unhealthy lifestyle is an increased amount of adipose tissue, which can influence body composition and cardiovascular status [4]. A high amount of adipose tissue is associated with higher inflammation, which in turn, may negatively affect the cardiovascular system and increase the risk of cardiovascular disorders as well as inhibit the formation of new neurons [5–7]. Fortunatley, these morbidities can be reduced by preventive interventions [8]. Therefore, approaches such as increasing physical activity and consumption of healthy diets have been identified as efficient approaches in children to decrease the incidence of these chronic morbidities. These approaches also have far reaching effects on the society, not just in terms of improving health but also in the reduction of cost of care [8–10].

The World Health Organisation (WHO) has recommended that, at least, 60 min of PA carried out daily is necessary for children [11]. While encouraging children to be more physically active and eating healthy is to a large extent a responsibility of the parents, the school system can play a significant role in stimulating children to adopt a healthy lifestyle. Indeed, several education programs have been developed in schools towards this goal [12, 13]. When such programs are included during school hours—and even included in the school curriculum—they have the advantage that the included health promotion activities are accessible to all children from different socioeconomic backgrounds [14]. Numerous studies have found a reduction in body mass index and fat mass with increased PA, and that these positive effects are even maintained after years following such an intervention [15–17].

Earlier studies have analysed the effects of a combined daily PA and healthy nutrition; however, the results of these studies vary widely and, in some cases, are even contradictory [18]. While some earlier studies reported no effect, others have observed that a combination of daily PA and healthy nutrition provides the best effect towards weight reduction and cardiovascular fitness improvement [18–21]. Therefore, the present study simultaneously evaluates the effects of a synergy of a PA educational program and healthy/unhealthy nutritional habits. The aim of the HOPP study is to evaluate whether nutritional habits in combination with an additional 45-min of daily moderate-to-vigorous PA during school hours positively affect anthropometric characteristics in elementary school children.

## Methodology

### Participants

A total of 917 fourth graders from nine different schools from the south–east of Norway took part in this study. They were divided into an intervention group (*n* = 614) and a control group (*n* = 303). The two groups had similar socioeconomic status. The control group had a traditional education program, which included a compulsory 90-min PA per week. An additional daily 45-min active learning session was introduced to the intervention group. Doing these sessions, teachers gave lectures on academic subjects while the children were engaged in moderate to vigorous physical activity in the outdoor playground. For example, in the mathematics relay activity, the children received a number (e.g. 54). They then had to run to four different stations/places to find the correct numbers between 1 and 9, and they kept on running and adding the numbers until a total of 54 was obtained.

The intervention program started in 2015 for children in the 1–6th grade. In both the groups, baseline measurements were performed followed by annual tests. Assessed were several parameters, including anthropometry, body composition, fitness, and PA levels (see details below).

All 4th graders in 2015 (born 2005) performed baseline measurements. In 2016, children born in 2005 (now 5th graders) performed the test again in order to get one-year follow-up data on the same children. In 2016, an additional group of 4th graders (born 2006) had one-year intervention follow-up. Accordingly, 4th graders in the intervention group in 2017 (born 2007) had two-years intervention. And finally, 4th graders in 2018 (born 2008) were enrolled in the intervention group for 3 years before the questionnaire was administered (Table 1). This implies that the effect of diet on anthropometric variables included both one-year effect for the class of 2005, and secular trends for the classes of 2006, 2007 and 2008.

**Table 1:** The number of participants for each grade and year.

*n* = 389		*n* = 759		*n* = 381		*n* = 351	
IG *n* = 264	CG *n* = 125	IG *n* = 493	CG *n* = 266	IG *n* = 264	CG *n* = 117	IG *n* = 248	CG *n* = 103
		4th-206 *n* = 229	4th-2006 *n* = 141				
4th-2005 *n* = 264	4th-2005 *n* = 125	5th-2005 *n* = 264	5th-2005 *n* = 125	4th-2007 *n* = 264	4th-2007 *n* = 117	4th-2008 *n* = 248	4th-2008 *n* = 103
2015		2016		2017		2018	

The numbers are estimated based on enrolment in 2015. (Fredriksen et al. 2017. BMC Public Health).

*IG* intervention group, *CG* control group.

The parents of all children were informed about the procedure and signed informed consent was obtained from all participants. A complete description of the study protocol is given in Fredriksen et al. [14].

### Measured variables

Measurement of anthropometry, body composition, fitness and PA levels were carried out. Muscle mass (kg), bone mass (kg) and body mass (kg) were assessed using a bio-impedance Tanita scale (Tanita MC-980MA, Tokyo, Japan) [22]. The body mass index (BMI) for the children was calculated from the body mass divided by the body height/squared and then adjusted for age and sex (isoBMI). In this paper, we used the isoBMI instead of the *z* score. The isoBMI depicts the children’s BMI in relation to the adult BMI of 25, 30 and 35 (overweight, obesity, morbid obesity), while the *z* score is only another way of ranking the children’s BMI. There may, therefore, be reasons to use isoBMI when the children’s BMI is to be assessed in relation to overweight and obesity, since isoBMI is based on absolute values. Cut-off values for *z* score only show differences for different datasets, and are not related to morbidity, as is the isoBMI. Thus, based on the isoBMI, the children were categorized into thin, normal weighted and overweight [23, 24].

In addition, a nutrition questionnaire (Ungkost2000) was used for the fourth graders [25]. This is a nation-wide (Norwegian) used questionnaire which assessed the nutritional characteristics of children. This nutritional survey includes 18 multiple-choice questions (age, sex, diet and body weight) [26]. Children were also asked if they took part in regular mealtimes such as breakfast, lunch and dinner. Finally, questions related to the frequency of soft drinks consumption (with- and without- sugar), and the frequency of the weekly consumption of various foods, including vegetables, bread, dairy products, fish, fast-food, sweets and vitamins were also included [26].

### Statistical procedure

All data were analysed using IBM SPSS v. 23 (IBM, Armonk, NJ, USA) statistical software. One-year effect on the class of 2005 was analysed using student *t* test. The secular trends were analysed using Linear mixed model (LMM) with a restricted maximum likelihood approach which is suitable for analyses of multilevel clustered data. A model using nine schools as a random factor and classes nested within the schools was used to create a random slope in the LMM procedure. The fixed factors were specified as the interaction between all fourth grader groups, the categorical questionnaires and the intervention/control groups. The reference points for the statistical analysis were specified as following: (i) Fourth graders in 2015 (baseline group); and (ii) None or rare intake of some specific types of food or beverage (according to the questionnaires); and (iii) control group of all fourth graders. Statistical significance was achieved when the target variables for a specific questionnaire was in relation to all three reference points. All target variables were metric and included the weight, muscle mass, fat mass and bone mass. Data are presented as mean ± SD.

## Results

A prospective study was conducted during 2015–2018 in nine different schools in the south-east of Norway. Details of the dietary intake and anthropometric variables in the control and intervention groups at baseline and over time is presented in Table 2.

**Table 2:** The table displays values from baseline, 1, 2- and 3-year secular trends for anthropometric variables for both intervention and control group.

	Baseline		1-year		2-years		3-years	
	Intervention	Control	Intervention	Control	Intervention	Control	Intervention	Control
	(*n* = 176)	(*n* = 98)	(*n* = 144)	(*n* = 96)	(*n* = 183)	(*n* = 62)	(*n* = 111)	(*n* = 47)
Age	9.7 (0.3)	10 (0.3)	9.7 (0.3)^a^	10.1 (0.3)^a^	10.5 (0.8)	11.5 (0.5)	10.4 (0.5)	10.1 (0.8)
BMI	16.9 (2.3)	16.7 (1.8)	17.7 (2.2)^ab^	16.8 (1.6)^a^	17.4 (2.3)^a^	16.5 (2.2)^a^	17.5 (2.4)	16.9 (1.8)
BMI (overweight)	20.7 (0.8)	20.8 (0.6)	20.9 (0.9)	20.7 (0.5)	188.8 (0.6)	M.D.	18.9 (0.6)	18.9 (0.9)
Weight	33.1 (5.8)	33.7 (5.6)	34.6 (5.5)^b^	33.5 (4.2)	33.8 (5.7)	32.5 (6.0)	34.1 (6.1)	33.9 (5.0)
Weight (overweight)	42.3 (3.4)	44.7 (2.6)	40.4 (2.9)	41.7 (2.5)	36.8 (3.8)	M.D.	38.4 (4.5)	39.4 (3.4)
Height	139.4 (5.9)	141.5 (6.3)	139.4 (6.2)	140.9 (5.1)	139.4 (6.0)	139.9 (5.8)	139.7 (6.7)	141.5 (5.7)
Muscle mass	24.5 (3.5)	25.3 (3.5)	25.3 (3.4)	25.1 (2.7)	24.7 (3.3)	24.5 (3.5)	24.8 (3.4)	25.6 (3.0)
Bone mass	7.1 (2.6)	6.9 (2.2)	7.8 (2.7)	6.9 (2.0)	7.7 (3.0)^a^	6.8 (2.7)^a^	7.6 (3.0)	6.8 (2.3)

Values are mean ± SD.

*M.D.* missing data

^a^Significant differences between intervention and control group.

^b^Significant changes associated with intervention time (compared to baseline).

### Effect of PA intervention on anthropometry

The two groups (intervention and control) of fourth graders in 2016 differ in age and BMI while in 2017 the fourth graders differed in BMI and bone mass. There were no other observables differences between the two groups. Further, observable differences in BMI and weight were seen between 2015 fourth graders, which represent the baseline intervention group, and the 2016 fourth graders that had completed a 1-year PA intervention: a higher BMI and weight was seen in the 1-year intervention group. Fourth graders from 2017 (with 2-years intervention) or 2018 fourth graders (with 3-years intervention) did not differ significantly from 2015 fourth graders (baseline intervention group) in BMI, weight, muscle mass and bone mass (Table 2).

### Effects of combined PA and nutritional intake on anthropometry

A daily PA combined with a regular intake of vegetables led to beneficial effects on muscle and bone mass. Daily PA and 4–6 times in a week vegetables intake was shown to positively influence muscle mass (3-years PA, *p* = 0.011 and 2-years PA, *p* = 0.028) and bone mass (3-years PA, *p* = 0.015). Similarly, four-times a day vegetable intake was shown to positively influence muscle mass (3-years PA, *p* = 0.015 and 2-years PA, *p* = 0.027) and bone mass (3-years PA, *p* = 0.019). Children with only regular daily intake of vegetables had less increase in muscle and bone mass compared to when both regular intake of vegetables and additional daily PA were carried out in combination. This beneficial effect was independent of the intervention duration. Furthermore, a combination of daily PA and regular intake of wholegrain bread was associated with lower weight and fat mass and a higher muscle mass as compared to a daily PA without a daily intake of wholegrain bread. Daily PA and 4–6 times in a week wholegrain bread intake was shown to negatively influence the weight (2-years PA, *p* = 0.015) and fat mass (2-years PA, *p* = 0.012). Daily PA and three-times a day wholegrain bread intake positively influenced muscle mass (3-years PA, *p* = 0.037 and 2-years PA, *p* = 0.04) (Tables 3, 4).

**Table 3:** The table display the effects of a daily physical activity and vegetable or sugar intake on anthropometric parameters in school children.

Questionnaires (*n* = 904)	Groups (Intervention, *n* = 603; control, *n* = 300)	Muscle mass				Bone mass			
		Baseline	1-year	2-years	3-years	Baseline	1-year	2-years	3-years
		(*n* = 263)	(*n* = 240)	(*n* = 243)	(*n* = 158)	(*n* = 263)	(*n* = 240)	(*n* = 242)	(*n* = 158)
Rarely (*n* = 77)	Intervention	27.0 (1.9)	24.8 (3.5)	24.7 (3.1)	23.6 (2.8)	1.56 (0.1)	1.41 (1.84)	1.43 (0.14)	1.33 (0.13)
	Control	25.1 (4.6)	24.2 (2.9)	23.9 (3.3)	M.D.	M.D.	1.39 (0.17)	1.35 (0.07)	M.D.
1–3 times a month (*n* = 65)	Intervention	24.8 (4.3)	24.1 (2.9)	25.7 (4.0)	23.6 (3.3)	1.41 (0.24)	1.37 (0.16)	1.45 (0.19)	1.36 (0.19)
	Control	25.0 (3.2)	25.5 (3.0)	26.4 (3.0)	25.1 (1.4)	1.41 (0.27)	1.46 (0.17)	1.53 (0.15)	1.46 (0.05)
1–3 times a week (*n* = 174)	Intervention	24.7 (3.9)	26.5 (3.6)	25.0 (3.9)	24.4 (4.2)	1.43 (0.23)	1.49 (0.19)	1.40 (0.21)	1.38 (0.23)
	Control	M.D.	24.6 (2.4)	M.D.	25.5 (3.5)	1.44 (1.9)	1.39 (1.40)	M.D.	1.44 (0.2)
4–6 times a week (*n* = 127)	Intervention	24.2 (3.3)	25.1 (3.3)	25.0 (2.9)^a^	25.2 (3.4)^a^	1.37 (0.18)	1.41 (0.19)	1.43 (0.16)	1.44 (0.16)^a^
	Control	25.7 (3.9)	25.6 (3.4)	23.7 (4.0)	25.0 (3.3)	1.45 (0.22)	1.45 (0.2)	1.36 (0.2)	1.4 (0.17)
2 times a day (*n* = 58)	Intervention	25.3 (4.7)	24.5 (2.8)	24.7 (3.8)	24.3 (1.7)	1.45 (0.27)	1.36 (1.59)	1.4 (0.18)	1.35 (0.1)
	Control	24.9 (3.4)	26.4 (3.2)	23.9 (4.2)	23.7 (1.0)	1.41 (0.18)	1.46 (0.17)	1.35 (0.19)	1.3 (0.05)
3 times a day (*n* = 62)	Intervention	26.0 (4.0)	25.2 (5.1)	23.4 (3.2)	24.6 (2.9)	1.45 (0.24)	1.43 (0.28)	1.31 (0.18)	1.41 (0.15)
	Control	25.3 (3.8)	24.8 (2.0)	25.1 (2.9)	28.1 (1.0)	1.41 (0.22)	1.40 (0.1)	1.38 (0.15)	1.56 (0.5)
4 or more times a day (*n* = 121)	Intervention	24.3 (3.2)	25.8 (2.9)	24.7 (3.0)^a^	25.1 (3.4)^a^	1.35 (0.18)	1.48 (0.16)	1.4 (0.17)	1.4 (0.17)^a^
	Control	26.4 (3.3)	24.7 (1.3)	23.8 (4.4)	25.4 (2.7)	1.49 (0.19)	1.45 (0.05)	1.37 (0.21)	1.41 (0.17)

All variables are displayed as mean ± SD.

*M.D.* missing data

^a^Significant interaction between nutrition and physical activity on anthropometric outcomes.

**Table 4:** The table display the effects of physical activity and wholegrain bread intake on anthropometric parameters in school children.

Questionnaires (*n* = 917)	Groups (Intervention, *n* = 614; control, *n* = 303)	Weight			Fat mass			Muscle mass		
		Baseline	2-years	3-years	Baseline	2-years	3-years	Baseline	2-years	3-years
		(*n* = 274)	(*n* = 245)	(*n* = 158)	(*n* = 263)	(*n* = 243)	(*n* = 158)	(*n* = 263)	(*n* = 243)	(*n* = 158)
Rarely (*n* = 158)	Intervention	31.3 (5.5)	34.4 (5.3)	33.3 (7.2)	6.5 (2.2)	8.1 (3.3)	8.1 (4.0)	23.4 (3.8)	24.8 (2.6)	23.8 (3.6)
	Control	32.9 (7.0)	31.1 (5.1)	32.0 (3.3)	6.6 (2.5)	6.1 (2.5)	5.6 (0.9)	24.8 (4.6)	24.4 (3.0)	24.9 (2.8)
1–3 times a month (*n* = 194)	Intervention	32.4 (4.7)	33.6 (7.0)^a^	35.8 (5.8)	6.3 (1.7)	8.1 (3.9)^a^	8.8 (2.8)	24.7 (3.2)	24.2 (3.5)^a^	25.6 (3.6)
	Control	31.3 (5.3)	40.2 (6.4)	36.6 (7.2)	6.0 (1.9)	10.0 (4.0)	8.2 (4.6)	23.6 (3.2)	25.8 (2.5)	26.9 (2.9)
1–3 times a week (*n* = 205)	Intervention	32.6 (5.6)	33.5 (5.3)	32.7 (5.4)	7.3 (3.2)	7.4 (2.7)	7.0 (2.4)	24.3 (2.4)	24.6 (3.2)	24.3 (3.1)
	Control	35.1 (4.8)	34.7 (7.1)	36.5 (5.2)	7.7 (2.1)	7.9 (3.2)	7.5 (2.6)	25.8 (3.09)	23.4 (4.3)	27.4 (2.6)
4–6 times a week (*n* = 118)	Intervention	35.7 (5.5)	32.8 (5.3)^a^	34.2 (6.3)	8.0 (2.8)	6.7 (1.8)^a^	7.7 (3.0)	26.2 (3.2)	24.7 (3.6)	25.1 (3.5)
	Control	33.0 (6.5)	34.3 (6.7)	31.7 (2.9)	6.7 (2.8)	7.6 (2.7)	6.5 (0.9)	24.9 (3.7)	25.1 (4.1)	23.8 (2.3)
2 times a day (*n* = 67)	Intervention	33.0 (6.0)	33.9 (7.3)	34.7 (5.3)	7.2 (2.1)	8.2 (3.2)	7.2 (2.6)	24.3 (4.1)	24.4 (4.2)	24.6 (2.5)
	Control	34.8 (5.2)	29.5 (2.1)	36.1 (5.6)	6.9 (1.8)	4.9 (0.9)	8.3 (3.5)	26.3 (3.4)	23.2 (1.7)	26.3 (2.2)
3 times a day (*n* = 48)	Intervention	33.4 (7.7)	36.3 (5.4)	36.1 (8.4)	8.0 (3.8)	8.5 (2.8)	6.6 (3.1)	23.8 (4.3)	26.3 (2.6)^a^	27.9 (5.0)^a^
	Control	35.6 (6.7)	28.5 (3.6)	M.D.	7.9 (2.5)	5.4 (1.2)	M.D.	26.2 (4.4)	21.3 (2.5)	M.D.
4 or more times a day (*n* = 56)	Intervention	33.0 (3.9)	34.0 (5.4)	34.0 (7.2)	6.6 (1.8)	7.2 (2.4)	7.9 (3.6)	25.0 (2.9)	25.5 (3.5)	24.7 (4.1)
	Control	36.0 (3.6)	M.D.	35.6 (9.3)	7.4 (1.3)	M.D.	8.4 (3.5)	26.7 (2.9)	M.D.	25.7 (5.5)

All variables are displayed as mean ± SD.

*M.D.* missing data

^a^significant interaction between nutrition and physical activity on anthropometric outcomes.

### Effects of combination of PA and unhealthy nutritional intake on anthropometry

Daily PA combined with regular intake of unhealthy fast food was associated with lower muscle and bone mass as compared to a daily PA without the intake of fast food. Daily PA and 1–3 times in a week fast food intake negatively influenced muscle mass (3-years PA, *p* = 0.034 and 2-years PA, *p* = 0.038) and bone mass (3-years PA, *p* = 0.010 and 2-years PA, *p* = 0.015). Furthermore, muscle and bone mass decreased with higher consumption of soft drinks with sugar regardless of additional daily PA. Daily PA and 4–6 times in a week soft drinks with sugar intake negatively influenced muscle mass, (2-years PA, *p* = 0.005) and bone mass (2-years PA, *p* = 0.007) and 1–3 times a day intake of soft drinks with sugar was shown to negatively influence muscle mass (2-years PA, *p* = 0.037). However, despite increased regular intake of soft drinks with sugar, daily PA led to reductions in fat mass. A decrease of muscle and bone mass was also seen by a regular intake of soft drinks without sugar. But our data suggest that a combination of daily PA and a regular intake of soft drinks without sugar was associated with a lower weight and fat mass (3-years PA, *p* = 0.035). Daily PA and 4–6 times in a week soda without sugar (soda light) intake negatively influenced weight (2-years PA, *p* = 0.002 and 1-year PA, *p* = 0.012) and muscle mass (2-years PA, *p* = 0.023) and fat mass (2-years PA, *p* = 0.001 and 1-year PA, *p* = 0.008) (Tables 5–7).

**Table 5:** The table display the effects of physical activity and fast food intake on the anthropometric parameters of school children.

Questionnaires (*n* = 904)	Groups (Intervention, *n* = 604; control, *n* = 300)	Muscle mass			Bone mass		
		Baseline	2-years	3-years	Baseline	2-years	3-years
		(*n* = 263)	(*n* = 243)	(*n* = 158)	(*n* = 263)	(*n* = 242)	(*n* = 158)
Rarely (*n* = 223)	Intervention	24.6 (3.4)	24.3 (3.2)	23.8 (3.3)	1.39 (0.2)	1.39 (0.16)	1.36 (0.16)
	Control	26.0 (3.5)	24.2 (3.5)	24.8 (2.4)	1.42 (0.19)	1.38 (0.19)	1.4 (0.13)
1–3 times a month (*n* = 367)	Intervention	24.3 (3.6)	24.8 (3.4)	25.6 (3.4)	1.38 (0.2)	1.4 (0.18)	1.45 (0.17)
	Control	25.4 (3.8)	24.2 (3.7)	25.3 (3.3)	1.43 (0.22)	1.35 (0.19)	1.41 (0.18)
1–3 times a week (*n* = 261)	Intervention	25.0 (3.7)	24.7 (2.9)^a^	24.3 (3.1)^a^	1.43 (0.2)	1.4 (0.18)^a^	1.38 (0.17)^a^
	Control	23.7 (2.2)	26.2 (3.4)	27.6 (3.2)	1.34 (0.09)	1.49 (0.15)	1.56 (0.15)
4–6 times a week (*n* = 24)	Intervention	23.5 (2.5)	24.7 (2.1)	22.9 (4.7)	1.32 (0.17)	1.4 (0.1)	1.27 (0.29)
	Control	M.D.	24.3 (3.0)	M.D.	M.D.	1.43 (0.11)	M.D.

All variables are displayed as mean ± SD.

*M.D.* missing data

^a^Significant interaction between nutrition and physical activity on anthropometric outcomes.

**Table 6:** The table display the effects of physical activity and beverage with sugar intake on the anthropometric parameters of school children.

Questionnaires (*n* = 903)	Groups (Intervention, *n* = 603; control, *n* = 300)	Muscle mass			Bone mass		
		Baseline	1-year	2-years	Baseline	1-year	2-years
		(*n* = 263)	(*n* = 240)	(*n* = 242)	(*n* = 263)	(*n* = 240)	(*n* = 243)
Rarely (*n* = 302)	Intervention	22.8 (4.1)	28.9 (6.6)	26.0 (5.7)	1.3 (0.2)	1.6 (0.3)	1.41 (0.28)
	Control	21.7 (2.6)	M.D.	23.5 (0.1)	1.3 (0.1)	M.D.	1.4 (0.1)
1–3 times a month (*n* = 250)	Intervention	24.9 (3.7)	26.8 (5.1)	25.2 (3.7)	1.4 (0.2)	1.5 (0.3)	1.41 (0.19)
	Control	24.9 (3.2)	27.6 (4.7)	24.5 (3.1)	1.4 (0.2)	1.54 (0.23)	1.38 (0.16)
1–3 times a week (*n* = 241)	Intervention	24.9 (4.7)	27.2 (4.2)	23.8 (3.7)	1.4 (0.3)	1.5 (0.2)	1.35 (0.2)
	Control	26.5 (4.1)	26.7 (3.5)	25.3 (4.6)	1.5 (0.2)	1.51 (0.22)	1.4 (0.25)
4–6 times a week (*n* = 43)	Intervention	25.8 (3.8)	27.3 (3.1)^a^	26.0 (3.6)^a^	1.5 (0.2)	1.5 (0.2)^a^	1.46 (0.18)^a^
	Control	M.D.	27.6 (3.8)	24.7 (3.3)	M.D.	1.55 (0.22)	1.36 (0.16)
1–3 times a day (*n* = 28)	Intervention	25.3 (3.7)	26.6 (4.4)	25.8 (3.8)^a^	1.4 (0.2)	1.5 (0.2)	1.46 (0.2)
	Control	26.7 (3.9)	26.6 (4.8)	24.3 (4.3)	1.5 (0.2)	1.49 (0.26)	1.37 (0.21)

All variables are displayed as mean ± SD

*M.D.* Missing data

^a^Significant interaction between nutrition and physical activity on anthropometric outcomes.

**Table 7:** The table display the effects of physical activity and beverage without sugar intake on the anthropometric parameters of school children.

Questionnaires (*n* = 917)	Groups (Intervention, *n* = 614; control, *n* = 303)	Weight				Fat mass				Muscle mass				Bone mass			
		Baseline	1-year	2-years	3-years	Baseline	1-year	2-years	3-years	Baseline	1-year	2-years	3-years	Baseline	1-year	2-years	3-years
		(*n* = 274)	(*n* = 203)	(*n* = 245)	(*n* = 158)	(*n* = 263)	(*n* = 240)	(*n* = 243)	(*n* = 158)	(*n* = 263)	(*n* = 240)	(*n* = 243)	(*n* = 158)	(*n* = 263)	(*n* = 240)	(*n* = 242)	(*n* = 158)
Rarely (*n* = 399)	Intervention	27.3 (4.5)	M.D.	36.55 (6.8)	M.D.	5.2 (0.7)	M.D.	8.4 (4.1)	M.D.	20.8 (3.8)	M.D.	26.6 (2.6)	M.D.	1.2 (0.3)	M.D.	1.5 (80.1)	M.D.
	Control	M.D.	M.D.	25.9 (0.1)	M.D.	M.D.	M.D.	4.7 (0.1)	M.D.	M.D.	M.D.	25.3 (0.1)	M.D.	M.D.	M.D.	1.5 (0.1)	M.D.
1–3 times a month (*n* = 199)	Intervention	34.6 (5.4)	34.2 (5.4)	35.2 (6.9)	33.7 (6.2)	7.6 (2.8)	7.8 (2.4)	8.4 (3.3)	7.3 (2.6)	25.7 (3.0)	24.9 (3.5)	25.4 (3.8)	24.7 (3.8)	1.46 (0.2)	1.4 (0.2)	1.44 (0.2)	1.39 (0.2)
	Control	33.5 (6.1)	33.9 (4.8)	34.3 (6.3)	34.6 (4.3)	7.0 (2.5)	7.3 (2.6)	6.6 (2.9)	7.1 (1.9)	24.5 (3.9)	25.2 (2.7)	23.7 (3.7)	26.0 (2.8)	1.41 (0.22)	1.43 (0.2)	1.32 (0.2)	1.46 (0.2)
1–3 times a week (*n* = 231)	Intervention	33.3 (6.4)	35.1 (5.7)	32.3 (5.2)	34.4 (6.5)	7.3 (2.7)	8.1 (2.6)	7.1 (3.0)	7.7 (2.9)	24.4 (4.1)	25.6 (3.6)	23.7 (2.7)	24.7 (3.4)	1.39 (0.23)	1.44 (0.2)	1.36 (0.1)	1.4 (0.2)
	Control	31.6 (5.0)	33.6 (3.8)	34.2 (5.4)	35.2 (6.0)	6.5 (2.0)	6.8 (1.7)	7.1 (2.3)	7.1 (2.5)	24.0 (3.2)	25.3 (2.7)	25.6 (3.5)	26.6 (3.4)	1.37 (0.2)	1.43 (0.2)	1.45 (0.2)	1.48 (0.1)
4–6 times a week (*n* = 47)	Intervention	M.D.	32.9 (3.8)^a^	33.8 (4.2)^a^	34.3 (6.5)	M.D.	6.8 (1.5)^a^	7.3 (2.5)	8.0 (3.9)	M.D.	24.7 (2.7)	25.1 (2.2)^a^	24.8 (2.7)	M.D.	1.4 (0.2)	1.42 (0.1)^a^	1.43 (0.2)
	Control	35.5 (12.0)	34.7 (4.4)	45.6 (0.3)	M.D.	7.3 (4.8)	6.9 (2.0)	12.3 (2.3)	M.D.	26.6 (6.8)	26.2 (2.6)	31.6 (1.7)	M.D.	1.55 (0.4)	1.47 (0.1)	1.7 (0.1)	M.D.
1–3 times a day (*n* = 19)	Intervention	32.8 (5.7)	35.4 (6.7)	33.7 (5.5)	34.2 (6.1)	6.9 (2.5)	8.4 (3.7)	7.6 (2.8)	7.8 (3.3)	24.4 (3.4)	25.5 (3.4)	24.6 (3.2)	24.9 (3.4)	1.37 (0.19)	1.46 (0.2)	1.39 (0.2)	1.42 (0.2)
	Control	34.5 (5.4)	30.1 (4.4)	32.3 (5.3)	33.5 (5.5)	7.0 (2.1)	5.7 (1.7)	6.6 (2.7)	6.7 (2.6)	25.8 (3.4)	23.0 (3.2)	24.4 (2.9)	25.3 (3.3)	1.45 (0.2)	1.32 (0.2)	1.38 (0.2)	1.42 (0.2)

All variables are displayed as mean ± SD

*M.D.* missing data

^a^Significant interaction between nutrition and physical activity on anthropometric outcomes.

### Effect of PA and fruits/berries on anthropometry in children with overweight

In children with overweight, combination of additional daily PA and a regular intake of fruits and berries was associated with lower weight and fat mass. Daily PA and 4–6 times in a week intake of fruits and berries intake has shown to negatively influence weight (3-years PA, *p* = 0.023) and fat mass (3-years PA, *p* = 0.007); and two times a day fruits and berries intake negatively influenced weight (3-years PA, *p* = 0.018) and fat mass (3-years PA, *p* = 0.001). This was only observed when PA was carries out along with the ingestion of fruit/berries. However, this association was not seen when only the fruits and berries were consumed or only PA was carried out (Table 8).

**Table 8:** The table display the effects of physical activity and fruits and berries intake on the anthropometric parameters of school children.

Questionnaires (*n* = 123)	Groups (Intervention, *n* = 97; control, *n* = 26)	Weight		Fat mass	
		Baseline	3-year	Baseline	3-year
		(*n* = 31)	(*n* = 27)	(*n* = 30)	(*n* = 27)
Rarely (*n* = 14)	Intervention	41.0 (0.1)	40.9 (6.5)	9.4 (0.1)	10.9 (2.1)
	Control	45.5 (2.1)	33.6 (0.1)	M.D.	6.3 (0.1)
1–3 times a month (*n* = 20)	Intervention	38.5 (2.2)	40.5 (6.3)	8.9 (0.4)	9.7 (1.0)
	Control	M.D.	39.6 (2.7)	M.D.	93 (2.6)
1–3 times a week (*n* = 25)	Intervention	41.3 (3.5)	37.8 (2.8)	10.9 (1.4)	10.1 (1.1)
	Control	44.0 (0.1)	37.4 (0.1)	10.7 (0.1)	6.5 (0.1)
4–6 times a week (*n* = 15)	Intervention	44.5 (0.5)	30.0 (0.1)^a^	13.2 (1.3)	6.8 (0.1)^a^
	Control	43.0 (2.8)	40.3 (1.2)	11.9 (1.9)	9.3 (0.9)
2 times a day (*n* = 14)	Intervention	44.7 (0.9)	33.7 (4.8)^a^	12.9 (1.4)	8.4 (1.6)^a^
	Control	43.0 (0.1)	45.0 (0.1)	7.8 (0.1)	11.3 (0.1)

All variables are displayed as mean ± SD

*M.D.* missing data

^a^Significant interaction between nutrition and physical activity on anthropometric outcomes.

## Discussion

The aim of this study was to analyse the effects of combination of daily PA with an intake of healthy/unhealthy diet on different anthropometric parameters in school children. The main findings of this study are: (1) Daily PA and a regular healthy diet increases muscle and bone mass; (2) Unhealthy dietary practices such as consumption of fast food, soda with/without sugar decreases muscle- and bone- mass. despite carrying out daily PA; (3) Daily PA may counteract, to some extent, the negative effects of an unhealthy diet on weight and fat mass.; and (4) A combination of daily PA and a regular intake of fruits and berries is beneficial for lowering weight and fat mass in children with overweight.

In this study, a beneficial effect of only daily 45-min PA was not seen on body weight or body composition. Nevertheless, in the present study, fourth graders in 2016 that completed a 1-year PA intervention showed a higher BMI and body weight as compared to the baseline fourth graders in 2015. This is expected as the BMI tends to increase, especially in children with overweight and obesity. Earlier studies have reported contradictory findings regarding the effects of a physical education program on weight and body composition [27–31]. While some studies reported that school-based PA programs show positive effects such as maintenance of steady weight [27–30], others found only limited effects of a 2-year school-based physical activity intervention on body composition and cardiorespiratory fitness in 7-year-olds [31]. Yet another study reported that such programs could actually lead to weight reduction in children with overweight [28]. As these studies differ in design, number of involved schools and/or socioeconomical characteristics of the studied populations, it is difficult to compare their results. Similarly, the results of the intervention program carried out in the present study are difficult to compare with those seen in earlier studies due to the presence of many confounding factors in our study. One factor that may devalued the effects of the intervention program in our study, is that no true reference-baseline is present for each fourth graders group, while the 1-year intervention group in year 2015 were added as a reference-baseline point for the changes in the three other groups. Thus, without a baseline for each intervention group it becomes difficult to confirm if the same children improved over time, or if the duration of the intervention had an influence which could have abolished the positive effects of the intervention. This may explain why no significant effect was seen when only the physical activity was carried out.

Earlier studies have reported a positive effect of school hours PA on weight reduction. This beneficial effect was only seen, however, when it was carried out in combination with an intense after-school hours PA [32, 33]. A mandatory 45-min PA a day was performed as active learning task during school hours; however, in the present study the children did not get any instruction to engage in after-school hours PA. As the present study did not analyse after school hour PA, it may be possible that a “saturation” of PA was been achieved during school hours, and, consequently, less activity was performed after school leading to reductions in the net effect of the intervention. Also, it is possible that high economical, educational and public health standards in most developed countries, including Norway, play an important role in reducing the variability between intervention and control groups [34, 35]. It could also be possible that, in Norway, changes of the body composition in children are not detectable because both groups (intervention and control) experience the same positive effect on the health status, and hence a ceiling effect may present prior to the start of the study [36]. To achieve positive effects of such interventions, the interventions may have to last longer and/or the amount of PA to be increased.

Despite the current school-based intervention program not showing any effect on weight per se, a combination of daily PA and healthy nutritional behaviour showed promising effects on other anthropometric measures. For instance, a combination of daily 45-min PA and a daily intake of vegetables and wholegrain bread increased muscle and bone mass and decreased in weight. This was not seen with only a daily 45-min PA program or with only a daily intake of vegetables and wholegrain bread. Our results are in agreement with earlier studies that reported a positive effect of a school-based physical intervention in combination with a healthy diet on weight and other anthropometric variables [18–21]. These studies recommended that reductions in weight and improvements in body composition can occur via consumption of healthy foods during school hours as well as by encouraging children to eat more healthy foods at home [18, 19, 37–41]. Also, nutritional based intervention programs have reported that plant-derived foods are effective in lowering body weight [42]. Moreover, current evidence suggests that carrying out a daily PA routine and eating a healthy diet positively influence cognitive and mental function in children [6, 7]. Taken together, these data suggest that daily PA and a healthy diet has not only a positive effect on the anthropometry, but could also improve cognitive and academic outcomes in school children.

Earlier studies have reported beneficial effects of a school-based PA intervention on muscle and bone mass [43]. Also, several studies have highlighted the importance of a healthy diet and PA in achieving and maintaining a healthy body composition during growing up into adolescence and also later in adult life [43–48]. We also obtained similar results in our study (that is, PA intervention alone does not increase muscle and bones mass, but only when it is combined with a healthy diet (vegetables and wholegrain bread)). This could be an effect of a richer macro- and micro- nutritional intake, which provide necessary nutrient molecules for healthy growth. Also, our results could be an indicator of better health awareness of the parents in aspects such as maintenance of a healthy diet, and carrying out regular physical activity, even post-school/at home [49–51].

Our results show that an unhealthy diet (fast food, burger, kebab, soft drinks with sugar, etc.) was associated with a reduced muscle and bone mass. It could be that increased fast food consumption along with sedentary activity may have caused the reduction in muscle mass in our study. Since physical inactivity is associated with reduced muscular loading, it may theoretically also lead to reductions in bone mass. Comparable results that highlighted negative effects of an unhealthy diet on the musculoskeletal system have previously been reported [52]. Regular fast food intake was also associated with increases in body weight in these children. However, a combination of regular fast food intake with a daily 45-min PA removed negative effects of a sugar rich beverage intake and also led to reductions in weight and fat mass. These findings support the aim of the intervention program used in this study: towards maintenance of healthy weight and body composition in children who are not overly health aware.

A beneficial effect of a regular healthy diet of fruits and berries, in combination with a daily 45-min of PA, was seen on the body weight and fat mass in children with overweight in our study. However, daily 45-min PA or regular fruit and berries intake alone was not able to reduce body weight and fat mass. The importance of a daily PA in combination with a regular healthy diet (vegetables, wholegrain bread, fruits and berries) in weight reduction on children with overweight is in agreement with what has been reported from other studies [53–56].

In conclusion, a daily 45-min physical activity program in combination with a healthy diet shows promising effects on the children’s body composition by reducing weight and increasing muscle -and bone - mass. An implication of these findings may be that educational programs aimed at improvements in health status in children should give attention to intake of healthy foods as well as carrying out more physical activity. Further studies are necessary to assess how interventional programs, which combine school based physical educational program with an after-school hours PA and a healthy nutritional intervention part, can be implemented.

## References

[1] Leon BM, Maddox TM (2015). Diabetes and cardiovascular disease: epidemiology, biological mechanisms, treatment recommendations and future research. World J Diabetes.

[2] Sallis JF, Glanz K (2009). Physical activity and food environments: solutions to the obesity epidemic. The Milbank quarterly.

[3] Mason KE, Pearce N, Cummins S (2018). Associations between fast food and physical activity environments and adiposity in mid-life: cross-sectional, observational evidence from UK Biobank. Lancet Public Health.

[4] Kim J (2014). Experiences of health related lifestyles in high body fat but non-obese female college students in Korea. Osong Public Health Res Perspect.

[5] Gutierrez DA, Puglisi MJ, Hasty AH (2009). Impact of increased adipose tissue mass on inflammation, insulin resistance, and dyslipidemia. Curr Diab Rep.

[6] Davis CL, Tomporowski PD, Boyle CA, Waller JL, Miller PH, Naglieri JA (2007). Effects of aerobic exercise on overweight children’s cognitive functioning. Res Q Exerc Sport.

[7] Sharma. M, Saroha. P (2020). Descriptive study of role played by exercise and diet on brain plasticity. Int J Sport Health Sci.

[8] Pandita A, Sharma D, Pandita D, Pawar S, Tariq M, Kaul A (2016). Childhood obesity: prevention is better than cure. Diabetes, Metab Syndr Obes.

[9] Sharma B, Kim HY, Nam EW (2018). Effects of school-based health promotion intervention on health behaviors among school adolescents in North Lima and Callao, Peru. J Lifestyle Med.

[10] Ekwaru JP, Ohinmaa A, Tran BX, Setayeshgar S, Johnson JA, Veugelers PJ (2017). Cost-effectiveness of a school-based health promotion program in Canada: a life-course modeling approach. PLoS ONE.

[11] Füssenich LM, Boddy LM, Green DJ, Graves LEF, Foweather L, Dagger RM (2016). Physical activity guidelines and cardiovascular risk in children: a cross sectional analysis to determine whether 60 min is enough. BMC Public Health.

[12] Guerra PH, Nobre MRC, Silveira JACD, Taddei JADAC (2013). The effect of school-based physical activity interventions on body mass index: a meta-analysis of randomized trials. Clinics (Sao Paulo).

[13] Errisuriz VL, Golaszewski NM, Born K, Bartholomew JB (2018). Systematic review of physical education-based physical activity interventions among elementary school children. J Prim Prev.

[14] Fredriksen PM, Hjelle OP, Mamen A, Meza TJ, Westerberg AC (2017). The health Oriented pedagogical project (HOPP)—a controlled longitudinal school-based physical activity intervention program. BMC Public Health.

[15] Verjans-Janssen SRB, van de Kolk I, Van Kann DHH, Kremers SPJ, Gerards SMPL (2018). Effectiveness of school-based physical activity and nutrition interventions with direct parental involvement on children’s BMI and energy balance-related behaviors—a systematic review. PLoS ONE.

[16] Mei H, Xiong Y, Xie S, Guo S, Li Y, Guo B (2016). The impact of long-term school-based physical activity interventions on body mass index of primary school children—a meta-analysis of randomized controlled trials. BMC Public Health.

[17] Lavelle HV, Mackay DF, Pell JP (2012). Systematic review and meta-analysis of school-based interventions to reduce body mass index. J Public Health.

[18] De Bourdeaudhuij I, Van Cauwenberghe E, Spittaels H, Oppert J-M, Rostami C, Brug J (2011). School-based interventions promoting both physical activity and healthy eating in Europe: a systematic review within the HOPE project. Obes Rev.

[19] Gallotta MC, Iazzoni S, Emerenziani GP, Meucci M, Migliaccio S, Guidetti L (2016). Effects of combined physical education and nutritional programs on schoolchildren’s healthy habits. PeerJ..

[20] Angelopoulos PD, Milionis HJ, Grammatikaki E, Moschonis G, Manios Y (2009). Changes in BMI and blood pressure after a school based intervention: The children study. Eur J Public Health.

[21] Spiegel SA, Foulk D (2006). Reducing overweight through a multidisciplinary school-based intervention. Obesity..

[22] Haroun D, Croker H, Viner RM, Williams JE, Darch TS, Fewtrell MS (2009). Validation of BIA in obese children and adolescents and re-evaluation in a longitudinal study. Obesity (Silver Spring, Md).

[23] Kiuru E, Kokki H, Juvonen P, Lintula H, Paajanen H, Gissler M (2014). The impact of age and sex adjusted body mass index (ISO-BMI) in obese versus non-obese children and adolescents with cholecystectomy. In Vivo.

[24] Harrington DM, Staiano AE, Broyles ST, Gupta AK, Katzmarzyk PT (2013). BMI percentiles for the identification of abdominal obesity and metabolic risk in children and adolescents: evidence in support of the CDC 95th percentile. Eur J Clin Nutr.

[25] Andersen LF, Overby N, Lillegaard IT (2004). Intake of fruit and vegetables among Norwegian children and adolescents. Tidsskr Nor Laegeforen.

[26] Øverby NAL Ungkost-2000. Landsomfattende kostholdsundersøkelse blant elver i 4. -og 8. klasse i Norge. Oslo: Sosial- og helsedirektoratet, Avdeling for ernæring. 2002 40.

[27] Eather N, Morgan PJ, Lubans DR (2013). Improving the fitness and physical activity levels of primary school children: results of the Fit-4-Fun group randomized controlled trial. Prev Med.

[28] Hollar D, Messiah SE, Lopez-Mitnik G, Hollar TL, Almon M, Agatston AS (2010). Effect of a two-year obesity prevention intervention on percentile changes in body mass index and academic performance in low-income elementary school children. Am J Public Health.

[29] Donnelly JE, Greene JL, Gibson CA, Smith BK, Washburn RA, Sullivan DK (2009). Physical Activity Across the Curriculum (PAAC): a randomized controlled trial to promote physical activity and diminish overweight and obesity in elementary school children. Prev Med.

[30] Haerens L, Cerin E, Maes L, Cardon G, Deforche B, De (2008). Explaining the effect of a 1-year intervention promoting physical activity in middle schools: a mediation analysis. Public Health Nutr.

[31] Magnusson KT, Hrafnkelsson H, Sigurgeirsson I, Johannsson E, Sveinsson T (2012). Limited effects of a 2-year school-based physical activity intervention on body composition and cardiorespiratory fitness in 7-year-old children. Health Educ Res.

[32] Waters E, de Silva-Sanigorski A, Hall BJ, Brown T, Campbell KJ, Gao Y (2011). Interventions for preventing obesity in children. Cochrane Database Syst Rev.

[33] Dobbins M, De Corby K, Robeson P, Husson H, Tirilis D. School-based physical activity programs for promoting physical activity and fitness in children and adolescents aged 6–18. Cochrane Database Syst Rev. 2009;21:CD007651. 10.1002/14651858.CD007651. 10.1002/14651858.CD007651 19160341

[34] Müller AM, Alley S, Schoeppe S, Vandelanotte C (2016). The effectiveness of e-& mHealth interventions to promote physical activity and healthy diets in developing countries: a systematic review. Int J Behav Nutr Phys Act.

[35] Bauman A, Bull F, Chey T, Craig CL, Ainsworth BE, Sallis JF (2009). The international prevalence study on physical activity: results from 20 countries. Int J Behav Nutr Phys Act.

[36] Hruby A, Hu FB (2015). The epidemiology of obesity: a big picture. Pharmacoeconomics..

[37] Simons-Morton BG, Parcel GS, Baranowski T, Forthofer R, O’Hara NM (1991). Promoting physical activity and a healthful diet among children: results of a school-based intervention study. Am J Public Health.

[38] Perez-Rodrigo C, Aranceta J (2001). School-based nutrition education: lessons learned and new perspectives. Public Health Nutr.

[39] Wechsler H, Devereaux RS, Davis M, Collins J (2000). Using the school environment to promote physical activity and healthy eating. Prev Med.

[40] Xu F, Marchand S, Corcoran C, DiBiasio H, Clough R, Dyer CS (2017). A community-based nutrition and physical activity intervention for children who are overweight or obese and their caregivers. J Obes.

[41] Haerens L, Deforche B, Maes L, Stevens V, Cardon G, De Bourdeaudhuij I (2006). Body mass effects of a physical activity and healthy food intervention in middle schools. Obesity (Silver Spring, Md).

[42] Vaitkevičiūtė J, Petrauskienė A (2019). The associations between body mass index of seven- and eight-year-old children, dietary behaviour and nutrition-related parenting practices. Medicina (Kaunas).

[43] Lofgren B, Daly RM, Nilsson JA, Dencker M, Karlsson MK (2013). An increase in school-based physical education increases muscle strength in children. Med Sci Sports Exerc.

[44] Hervás G, Ruiz-Litago F, Irazusta J, Fernández-Atutxa A, Fraile-Bermúdez AB, Zarrazquin I (2018). Physical activity, physical fitness, body composition, and nutrition are associated with bone status in University students. Nutrients..

[45] Nguyen VH (2018). School-based exercise interventions effectively increase bone mineralization in children and adolescents. Osteoporos Sarcopenia.

[46] Fritz J, Rosengren BE, Dencker M, Karlsson C, Karlsson MK (2016). A seven-year physical activity intervention for children increased gains in bone mass and muscle strength. Acta Paediatr.

[47] Detter F, Nilsson J-Å, Karlsson C, Dencker M, Rosengren BE, Karlsson MK (2014). A 3-year school-based exercise intervention improves muscle strength—a prospective controlled population-based study in 223 children. BMC Musculoskelet Disord.

[48] Larsen MN, Nielsen CM, Helge EW, Madsen M, Manniche V, Hansen L (2018). Positive effects on bone mineralisation and muscular fitness after 10 months of intense school-based physical training for children aged 8-10 years: the FIT FIRST randomised controlled trial. Br J Sports Med.

[49] Jeffery AN, Voss LD, Metcalf BS, Alba S, Wilkin TJ (2005). Parents’ awareness of overweight in themselves and their children: cross sectional study within a cohort (EarlyBird 21). BMJ (Clinical research ed).

[50] Valmorbida JL, Goulart MR, Busnello FM, Pellanda LC (2017). Nutritional knowledge and body mass index: a cross-sectional study. Rev Assoc Med Bras (1992).

[51] Williamson J. Awareness of Physical. Activity Health Benefits can Influence. Participation and Dose. Sports Med. Rehabil J. 2016;1:1003.

[52] Vogel C, Parsons C, Godfrey K, Robinson S, Harvey NC, Inskip H (2016). Greater access to fast-food outlets is associated with poorer bone health in young children. Osteoporos Int.

[53] Gentile DA, Welk G, Eisenmann JC, Reimer RA, Walsh DA, Russell DW (2009). Evaluation of a multiple ecological level child obesity prevention program: switch what you do, view, and chew. BMC Med.

[54] Schroder KE (2010). Effects of fruit consumption on body mass index and weight loss in a sample of overweight and obese dieters enrolled in a weight-loss intervention trial. Nutrition..

[55] Llargues E, Franco R, Recasens A, Nadal A, Vila M, Pérez MJ (2011). Assessment of a school-based intervention in eating habits and physical activity in school children: the AVall study. J Epidemiol Community Health.

[56] Singh AS, Chin APMJ, Brug J, van Mechelen W (2009). Dutch obesity intervention in teenagers: effectiveness of a school-based program on body composition and behavior. Arch Pediatr Adolesc Med.

